# Reciprocal diversification in a complex plant-herbivore-parasitoid food web

**DOI:** 10.1186/1741-7007-5-49

**Published:** 2007-11-01

**Authors:** Tommi Nyman, Folmer Bokma, Jens-Peter Kopelke

**Affiliations:** 1 Faculty of Biosciences, University of Joensuu, FI-80101 Joensuu, Finland; 2 Department of Ecology and Environmental Science, Umeå University, SE-90187 Umeå, Sweden; 3 Forschungsinstitut Senckenberg, D-60325 Frankfurt am Main, Germany

## Abstract

**Background:**

Plants, plant-feeding insects, and insect parasitoids form some of the most complex and species-rich food webs. According to the classic escape-and-radiate (EAR) hypothesis, these hyperdiverse communities result from coevolutionary arms races consisting of successive cycles of enemy escape, radiation, and colonization by new enemy lineages. It has also been suggested that "enemy-free space" provided by novel host plants could promote host shifts by herbivores, and that parasitoids could similarly drive diversification of gall form in insects that induce galls on plants. Because these central coevolutionary hypotheses have never been tested in a phylogenetic framework, we combined phylogenetic information on willow-galling sawflies with data on their host plants, gall types, and enemy communities.

**Results:**

We found that evolutionary shifts in host plant use and habitat have led to dramatic prunings of parasitoid communities, and that changes in gall phenotype can provide "enemy-free morphospace" for millions of years even in the absence of host plant shifts. Some parasites have nevertheless managed to colonize recently-evolved gall types, and this has apparently led to adaptive speciation in several enemy groups. However, having fewer enemies does not in itself increase speciation probabilities in individual sawfly lineages, partly because the high diversity of the enemy community facilitates compensatory attack by remaining parasite taxa.

**Conclusion:**

Taken together, our results indicate that niche-dependent parasitism is a major force promoting ecological divergence in herbivorous insects, and that prey divergence can cause speciation in parasite lineages. However, the results also show that the EAR hypothesis is too simplistic for species-rich food webs: instead, diversification seems to be spurred by a continuous stepwise process, in which ecological and phenotypic shifts in prey lineages are followed by a lagged evolutionary response by some of the associated enemies.

## Background

One of the main challenges of biological research is to understand the evolutionary assembly and maintenance of complex, multitrophic food webs [1,2]. The classic escape-and-radiate (EAR) hypothesis [3] envisions the current remarkable diversity of plants and herbivorous insects [4,5] as a result of a cyclic coevolutionary process: a plant lineage that acquires a new defensive trait (e.g., a toxic chemical) becomes free to proliferate and rapidly divides into multiple descendant lineages [3,6]. Over time, the defenses of the new clade are overcome by some insect species, which now enter a vacant adaptive zone and diversify to exploit the species of the hitherto herbivore-free plant group [3,7]. A new cycle of diversification starts whenever a novel defense evolves in one of the plant lineages.

Although the EAR hypothesis was originally formulated in terms of plants and herbivores, it has recently been suggested that a concurrent EAR process operates between plant-feeding insects and their associated parasitoids [1,8]. Parasitic insects typically inflict heavy mortality on herbivore populations [9,10], and the specialized host use of both insect herbivores and parasitoids leads to the intriguing possibility that these hyperdiverse interaction networks are created "from within", that is, by diversifying effects that are transmitted or even amplified through many trophic levels. "Bottom-up" speciation cascades could result if diversification of plants spurs speciation of herbivores [5,7,11] that, in turn, leads to increased resource diversity for associated parasitoids [2,12,13]. "Top-down" diversifying forces could be equally important if parasitoids use plants as cues for finding their host insects; in such cases, an evolutionary shift to a novel host plant could provide "enemy-free space" for the herbivores [9,14,15]. Release from enemies could accelerate diversification in the herbivore lineage that, in turn, would create more possibilities for parasitoid speciation.

Diversifying selection exerted by natural enemies might similarly underlie the unusual diversity of many gall-inducing insect groups. The ability to induce galls on plants has evolved convergently in dozens of distantly related insect taxa and, as a result of spectacular adaptive radiations, many of these groups contain hundreds of species that differ markedly with respect to their host plant use and gall morphology [16,17]. Phylogeny-based comparative studies have demonstrated that galls represent "extended phenotypes" of the gallers, meaning that gall form and location is determined mainly by the galling insects and not by their host plants [17,18]. Because galler parasitoids have to penetrate a protective wall of modified plant tissue in order to gain access to their victims, Stone and Schönrogge [17] recently concluded that morphology-dependent parasitism remains the most plausible adaptive explanation for the diversification of gall form, but they also noted that the needed phylogenetic tests are lacking.

Despite its intuitive appeal, the EAR hypothesis has never been tested in a phylogenetic framework [8]. Therefore, we investigated how phylogenetic patterns of parasitism and diversification in gall-inducing sawflies belonging to the nematine subtribe Euurina (Hymenoptera: Tenthredinidae) conform to the predictions of the hypothesis. Euurina sawflies induce leaf folds or rolls, or various closed galls on willows (*Salix *spp.) and, at over 400 species, the subtribe includes over 10 times more species than its sister group with larvae that feed externally on leaves [19,20]. Their main sources of mortality are larvae of parasitoids (that feed on galler larvae) and parasitic inquilines (that consume gall tissues but kill the sawfly larvae in the process) [19,21]. In all, the associated enemy complex comprises nearly 100 species that belong to 17 families in four insect orders [19,22]. Sawfly gallers are particularly suited for studying the evolutionary assembly of complex food webs, because while each galler species is typically a specialist on a single willow species, all of the seven main types of closed galls can be found on multiple willow hosts [19,21]; this cross-replication of willows and galls makes it possible to tease apart the respective effects of host plants and gall phenotypes on the composition of the enemy community attacking each galler species.

## Results and discussion

**Figure 1:**
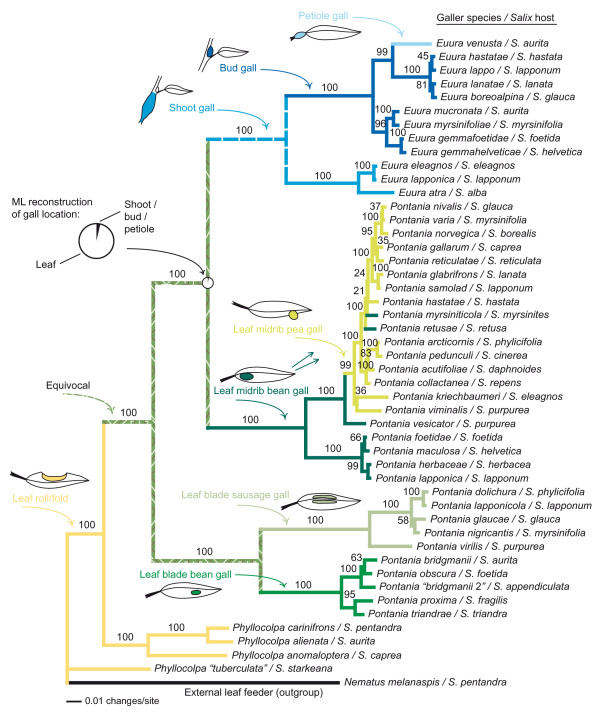
**Evolutionary diversification of gall morphology and host plant use in willow-galling sawflies**. The tree is according to a Bayesian phylogenetic analysis of 1 528 bp of DNA sequence data from two mitochondrial genes (see Methods), numbers above branches show posterior probabilities of clades. Host plants are indicated after the sawfly species names. Ancestral gall types were reconstructed using Accelerated transformations parsimony optimization (the ancestral state of the *Euura *clade is equivocal, but it was probably shoot galling [18]). The pie diagram at the node between *Euura *and their sister group shows the relative likelihood of different gall locations as reconstructed by maximum likelihood.

As a first step, we reconstructed the phylogenetic tree of willow gallers on the basis of DNA sequence data from two mitochondrial genes (see Methods). The strongly supported phylogeny (Figure 1) confirms earlier results that species inducing closed galls evolved from external-feeding sawfly lineages via leaf folders [18,20], and demonstrates that the galler community on any given willow species is a collection of sawflies inducing different galls that have colonized the host, or one of its ancestors, at different time intervals.

**Figure 2:**
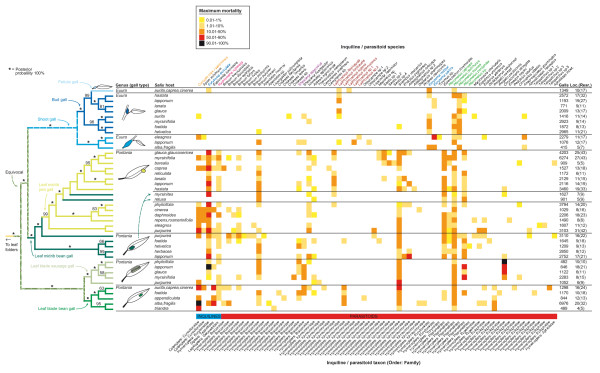
**Maximum rates of mortality inflicted by 72 inquiline and parasitoid species on 43 willow-galling sawfly species in relation to the phylogeny, gall morphology, and host plants of the gallers**. Sawfly names have been omitted, but their order is the same as in Figure 1. Each column in the plot represents one inquiline or parasitoid species, maximum rates of parasitism observed in extensive population rearings of each galler species [21,22] are indicated by the colour of the cells (see legend). See Additional file 1 for exact rates of parasitism. Enemy species and genera mentioned in the text are highlighted by a coloured font, and the taxon to which each parasite species belongs is indicated below the matrix (note that the wasp genus *Eurytoma *includes two parasitoids and an inquiline). Numbers of dissected galls, collection localities, and population rearings are given in the last two columns. Numbers above branches on the tree show Bayesian posterior probabilities (only values ≥ 50% shown, asterisks denote clades with a 100% posterior probability).

**Figure 3:**
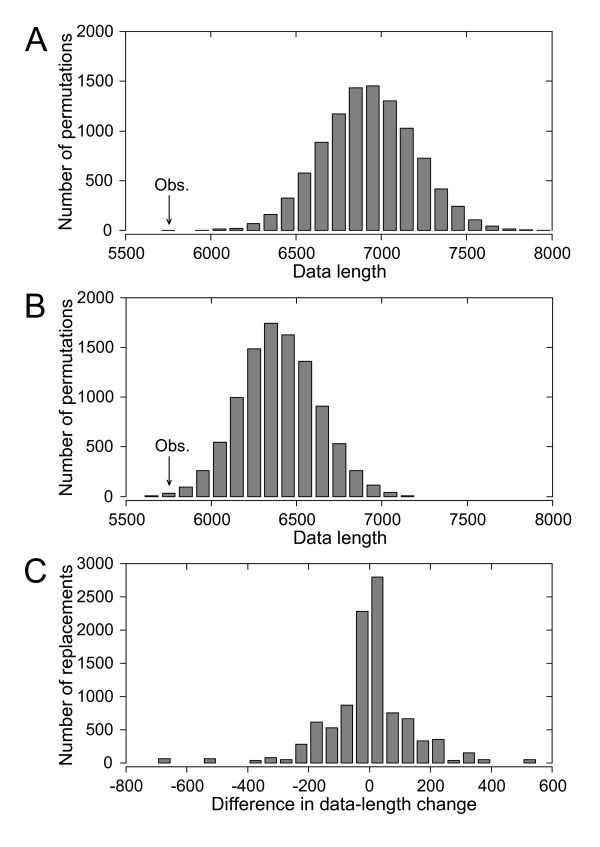
**Distributions of data lengths resulting from random permutations of galler enemy communities in relation to the galler phylogeny**. In (A) and (B), the arrow indicates the length of the observed quantitative parasitism data, as calculated on the basis of the galler topology in Figure 2. (A) Distribution of data lengths when enemy communities (rows) are permuted 10000 times across the whole galler phylogeny. (B) Distribution of data lengths when enemy communities are similarly permuted within gall-type groups. (C) Distribution of the difference in the absolute change in data length when the enemy complex of a galler species is replaced by those of two species from another gall type, of which only one also has a different host willow. Negative values of the difference indicate that the change in data length is smaller when the replacing species occurs on the same host plant (see Methods).

**Figure 4:**
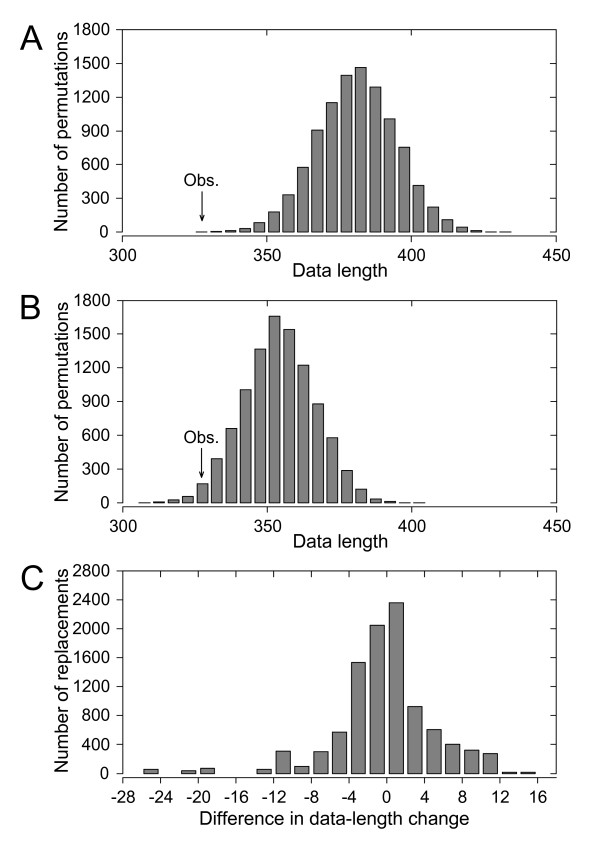
**Distributions of data lengths resulting from random permutations of galler enemy communities in relation to the galler phylogeny**. The histograms show the distributions of data lengths when qualitative (i.e., presence/absence) data on enemy species are used instead of the quantitative rates of mortality used in Figure 3. In (A) and (B), the arrow indicates the length of the observed qualitative parasitism data, as calculated on the basis of the galler topology in Figure 2. (A) Distribution of data lengths when enemy communities (rows) are permuted 10000 times across the whole galler phylogeny (p < 0.0001). (B) Distribution of data lengths when enemy complexes are similarly permuted within gall-type groups (p = 0.0158). (C) Distribution of the difference in the absolute change in data length when the enemy complex of a galler species is replaced by those of two species from another gall type, of which only one also has a different host willow (see Methods). Negative values of the difference indicate that the change in data length is smaller when the replacing species occurs on the same host plant (paired samples *t *test, *t *= -4.93, 95% c.i. -∞ to -0.174, one-tailed p < 0.001).

Contrasting the galler phylogeny with quantitative data on the mortalities inflicted by inquiline and parasitoid species shows that evolutionary changes in many different ecological traits can lead to full or partial release from natural enemies (Figure 2). Permutation tests demonstrate a strong correlation between galler phylogeny and species-level enemy communities (Figure 3A, p < 0.0001), which mainly follows from the fact that the largest differences in enemy communities occur among gall types, which are likewise strongly conserved with respect to the galler tree. Constraining permutations of species-level enemy communities to occur only within gall types also leads to longer data lengths (Figure 3B, p = 0.0022), but the increase tends to be less pronounced than in unconstrained randomizations. Within gall-type groups, enemy communities in many cases differ markedly among willow species, but a statistically significant host plant effect extends also across gall-type boundaries (Figure 3C; paired samples *t *test, *t *= -1.72, 95% c.i. -∞ to -0.104, one-tailed p = 0.043). These results are robust enough to be found also in randomization tests based on qualitative (presence/absence) data on enemy communities (Figure 4A–C).

Sawflies inducing identical galls on different host species are in many cases attacked by very different parasite assemblages (Figure 2), which indicates that directional selection coefficients imposed by natural enemies can be extremely strong during host plant shifts. For example, host-provided enemy-free space evidently can be found especially on several distantly related willow species that grow in sub-arctic and arctic-alpine habitats (e.g., *S. lapponum*, *S. reticulata*, and *S. myrsinites*) where, for example, the predominantly southern inquilines *Curculio crux *(Coleoptera: Curculionidae) and *Hydriomena ruberata *(Lepidoptera: Geometridae) occur only rarely (Figure 2). Conversely, leaf gallers on northern willows generally suffer from increased attack by the parasitic wasps *Shawiana lapponica *and *Lathrostizus flexicauda *(Hymenoptera: Ichneumonidae). Contrasting selection pressures caused by spatial and temporal variation in enemy communities [22-24] could explain why some prevalent and seriously lethal parasite species have been both lost and gained during the diversification of leaf-galling sawfly lineages (Figure 2).

**Figure 5:**
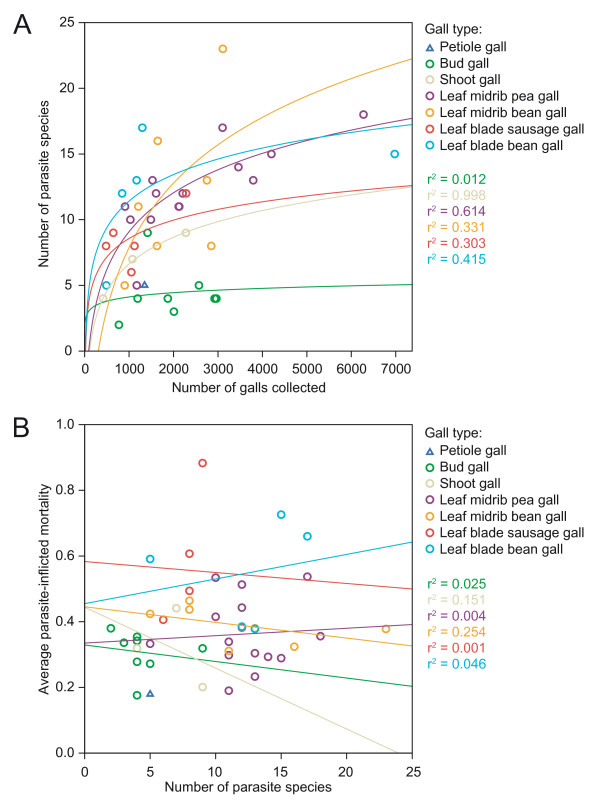
**Factors explaining numbers of enemy species and parasite-inflicted mortalities in individual galler species**. Each point in the plots represents one galler species. (A) Numbers of associated enemy species in relation to gall types and sample sizes. Lines show logarithmic regression curves for each gall type. (B) Average parasite-inflicted mortalities of individual galler species [22] in relation to their gall types and numbers of associated parasite species. Lines are not shown for petiole gallers that are represented by a single species (triangle). ANCOVA results for (A) and (B) are given in Tables 1 and 2, respectively.

However, the most dramatic shift in enemy communities coincides with the evolutionary transition from the ancestral condition of leaf galling to gall induction on shoots, buds, and petioles (Figures 1 and 2). This seemingly minor change in gall phenotype, which occurred at least six million years ago [25], led to a near-complete elimination of parasitic inquilines and to a coincident pruning of the parasitoid community (Figures 2 and 5A; Table 1), demonstrating that gall-inducing insects can find immediate and long-lasting "enemy-free morphospace" even in the absence of host plant shifts. The community plot shows that the novel gall types were subsequently tracked and colonized by parasitic lineages that apparently were derived mainly from the ancestral pool of enemies. In accordance with the EAR hypothesis, at least five probable cases of adaptive splitting along gall-type boundaries can be identified in the parasitic wasp genera *Lathrostizus *(Ichneumonidae), *Pteromalus *(Pteromalidae), and *Eurytoma *(Eurytomidae) (Figure 2), probably because successful attack on different galls requires specialized adaptations in the parasitoids' search behaviors and ovipositor structures [10,22,24].

**Table 1:** Effects of gall type and sample size (= number of galls collected) on the number of parasitoid species observed attacking each galler species

Source	Type III sum of squares	df	Mean square	*F*	p Value
Corrected model	592.188*	6	98.698	10.360	<0.001
Intercept	64.579	1	64.579	6.779	0.013
Number of galls	166.368	1	166.368	17.464	<0.001
Gall type	382.832	5	76.566	8.037	<0.001
Error	333.431	35	9.527		
Total	5046.000	42			
Corrected total	925.619	41			

df, degrees of freedom

Gall type was included as a fixed factor and sample size as a covariate in the ANCOVA model. Sample sizes were ln-transformed prior to the analysis, because logarithmic regressions produced more biologically realistic results and a better fit to the observed data (Figure 5A) than did linear regressions with untransformed sample sizes.

*R^2 ^= 0.640 (adjusted R^2 ^= 0.578).

The central prediction of the EAR hypothesis is that escapes from enemies trigger rapid radiations in prey lineages [3,6,8], but our phylogenetic results (Figures 1 and 2) directly contradict this proposition. Current estimates [19,21] of species numbers in the conspicuously parasite-poor *Euura *clade (ca. 100 spp.) are lower than those of their enemy-rich sister group composed of leaf midrib bean and pea gallers (ca. 150 spp.), and both of these clades contain more species than the monophyletic group comprising the enemy-rich leaf blade bean and sausage gallers (ca. 80 spp.). Additional comparative studies involving other insect taxa are needed to confirm this conflicting pattern, but it has been argued that the prediction of elevated speciation rates in enemy-free lineages has a weak theoretical basis [8], and our data suggest two explanations for the absence of notable speciation bursts. First, in complex food webs, release from some enemies can be quickly compensated by an increase in the severity of attack by the remaining ones, which is evidenced by a lack of association between numbers of parasite species and rates of parasite-inflicted mortality in comparisons across galler species (Pearson's *r *= 0.165, p = 0.291; Figure 5B, Table 2). Less obvious zero-sum games of survival are also possible, so that while overall rates of parasitism are slightly lower in *Euura *species than in leaf gallers, the benefit seems to be offset by an increased susceptibility to defence reactions on behalf of the host plants [19,22]. Second, if prey species are (like most plant feeding insects [4,5]) themselves resource specialists, a moderate enemy pressure might promote the prey's colonization of novel niches (e.g., plants [9,14,15]) and thus facilitate ecological speciation in prey lineages. This could especially be the case if geographical variation in enemy assemblages drives different populations of widespread prey species into using different resources.

**Table 2:** Effects of gall type and number of associated parasite species on average mortalities in individual galler species

Source	Type III sum of squares	df	Mean square	*F*	p Value
Corrected model	0.378*	6	0.063	3.834	0.005
Intercept	2.431	1	2.431	147.756	<0.001
Number of parasite species	0.001	1	0.001	0.040	0.843
Gall type	0.362	5	0.072	4.401	0.003
Error	0.576	35	0.016		
Total	20.437	42			
Corrected total	0.954	41			

Gall type was included as a fixed factor and number of parasite species as a covariate in the ANCOVA model (see Figure 5B). Mortalities were arcsine square root transformed prior to the analysis.

*R^2 ^= 0.397 (adjusted R^2 ^= 0.293).

## Conclusion

Coevolutionary studies on parasitoids and their prey commonly focus on physiological defenses and counterdefenses [26], but our results clearly show that ecological traits constitute a central part of the defensive arsenal of herbivorous insects. Prior to a niche shift, an evolving prey lineage must exhibit a polymorphism in resource use, which can be followed by quick fixation of one of the alternative states whenever different resources are associated with different enemy attack patterns. Furthermore, our finding that several parasitoid lineages have responded to gall-type divergence by adaptive speciation provides strong support for suggestions [1,2,8] that coevolutionary arms races have played an important role in the generation of the unusual diversity of herbivorous insects and parasitoids. Nevertheless, our data also indicate that in its classic form the EAR hypothesis is too simplistic to explain reciprocal diversification effects in complex food webs, in which escapes from enemies will tend to be too brief to lead to the predicted speciation bursts. Instead, the observed patterns of parasitism and diversification are consistent with a scenario of stepwise antagonistic coevolution: colonization of new ecological niches by prey lineages is being continuously driven by temporary relief from parasitism, after which an evolutionary response by some of the associated enemies returns mortalities to normal levels. In its complexity, the willow-galler-parasite food web is representative of many antagonistic networks in which specialized interactions link species across multiple trophic levels. A close integration of ecological and evolutionary research is clearly needed if the origins of such networks are to be fully understood.

## Methods

### Parasitism data and taxon sampling

Enemy rearing methods and collection localities of galler population samples have been described previously [21,22]. In brief, populations were sampled extensively throughout Europe between 1981 and 1998, and galls were opened to score the presence of galler or parasitoid/inquiline larvae. Enemy larvae were classified to preliminary morphospecies, and the identity of each morphospecies was determined by connecting them to adults emerging after hibernation. The severity of parasitism by each enemy species in each galler species was defined as the maximum observed level of mortality inflicted in the population samples (see Additional file 1 for exact rates of parasitism), which is a valid indicator of the potential importance of natural enemies [10]. To control for sampling effects, our phylogenetic analysis includes the 43 galler species (Table 3) that have parasitism data from at least four locations and a total sample size of over 400 galls. In accordance with previous results [18,25], we used the free-feeding nematine *Nematus melanaspis *and four leaf-folding or -rolling *Phyllocolpa *species as outgroups in the phylogenetic analyses.

**Table 3:** Taxa and samples used in the study, their gall types, willow hosts, and collection data

Genus (gall type)	Species	*Salix *host	Sample code	Specimen	Location and date	Collector
*Pontania *(Leaf blade sausage gall)	*dolichura*	*phylicifolia*	04151/Q1	Larva	Korvua, Finland, 9.vii.2004	T. Nyman
	*glaucae*	*glauca*	97194/Q2	Larva	Kilpisjärvi, Finland, 4.viii.1997	T. Nyman
	*lapponicola*	*lapponum*	05149/Z1	Larva	Nuorgam, Finland, 3.vii.2005	T. Nyman
	*nigricantis*	*myrsinifolia*	04236/Q3	Larva	Hintertux, Austria, 8.viii.2004	T. Nyman
	*virilis*	*purpurea*	05018/Y2	Larva	Kiesnitz, Oder, Germany, 18.v.2005	A. Liston

*Pontania *(Leaf blade bean gall)	*bridgmanii*	*aurita, caprea, cinerea*	97076/Q4	Larva	Mekrijärvi, Finland, 22.vii.1997	T. Nyman, A. Zinovjev
	*proxima*	*alba*, *fragilis*	04388/Q5	Larva	Oulu, Finland, 8.x.2004	T. Nyman
	*triandrae*	*triandra*	97455/Q6	Larva	Keminmaa, Finland, 29.vii.1997	T. Nyman
	*obscura*	*foetida*	04264/X6	Larva	Hintertux, Austria, 9.viii.2004	T. Nyman
	"*bridgmanii *2"	*appendiculata*	04125/X1	Larva	Vent-Sölden, Austria, 20.vi.2004	T. Nyman

*Pontania *(Leaf midrib bean gall)	*foetidae*	*foetida*	04263/Q9	Larva	Hintertux, Austria, 9.viii.2004	T. Nyman
	*herbaceae*	*herbacea*	98230/QX	Larva	Abisko, Sweden, 12.viii.1998	T. Nyman
	*lapponica*	*lapponum*	98308/R1	Larva	Abisko, Sweden, 16.viii.1998	T. Nyman
	*maculosa*	*helvetica*	04308/R2	Larva	Vent, Austria, 12.viii.2004	T. Nyman
	*retusae*	*retusa*	04243/R3	Larva	Hintertux, Austria, 8.viii.2004	T. Nyman
	*vesicator*	*purpurea*	04200/R5	Larva	Obergurgl, Austria, 5.viii.2004	T. Nyman

*Pontania *(Leaf midrib pea gall)	*acutifoliae daphnoides*	*daphnoides*	97490/R6	Larva	Latvia, 22.viii.1997	H. Roininen, A. Zinovjev
	*arcticornis*	*phylicifolia*	98328/R7	Larva	Kilpisjärvi, Finland, 18.viii.1998	T. Nyman
	*collactanea*	*repens*, *rosmarinifolia*	04124/R8	Larva	Blåvand, Denmark, 16.vi.2004	T. Nyman
	*gallarum*	*caprea*	98132/R9	Larva	Joensuu, Finland, 21.vii.1998	T. Nyman
	*glabrifrons*	*lanata*	98335/RX	Larva	Kilpisjärvi, Finland, 19.viii.1998	T. Nyman
	*hastatae*	*hastata*	01153/S1	Larva	Kilpisjärvi, Finland, 9.viii.2001	T. Nyman
	*kriechbaumeri*	*eleagnos*	04333/S2	Larva	Pertisau, Austria, 13.viii.2004	T. Nyman
	*myrsiniticola*	*myrsinites*	98364/S3	Larva	Kilpisjärvi, Finland, 23.viii.1998	T. Nyman
	*nivalis*	*glauca*, *glaucosericea*	98346/S4	Larva	Kilpisjärvi, Finland, 20.viii.1998	T. Nyman
	*norvegica*	*borealis*	98374/S5	Larva	Kilpisjärvi, Finland, 26.viii.1998	T. Nyman
	*pedunculi*	*cinerea*	98393/S6	Larva	Janakkala, Finland, 13.viii.1998	A. Zinovjev
	*reticulatae*	*reticulata*	04240/S7	Larva	Hintertux, Austria, 8.viii.2004	T. Nyman
	*samolad*	*lapponum*	98373/S8	Larva	Kilpisjärvi, Finland, 25.viii.1998	T. Nyman
	*varia*	*myrsinifolia*	98198/Z2	Larva	Joensuu, Finland, 20.vii.1998	T. Nyman
	*viminalis*	*purpurea*	04226/SX	Larva	Sölden, Austria, 7.viii.2004	T. Nyman

*Euura *(Bud gall)	*hastatae*	*hastata*	97497/T1	Larva	Kilpisjärvi, Finland, 13.viii.1997	T. Nyman
	*lanatae*	*lanata*	98024/T2	♀, *ex larva*	Kilpisjärvi, Finland, 15.viii.1997	T. Nyman
	*lappo*	*lapponum*	98362/T3	Larva	Kilpisjärvi, Finland, 23.viii.1998	T. Nyman
	*mucronata*	*aurita*	04385/T4	Larva	Oulu, Finland, 8.ix.2004	T. Nyman
	*gemmafoetidae*	*foetida*	04360/T5	Larva	Hintertux, Austria, 9.viii.2004	T. Nyman
	*boreoalpina*	*glauca*	98363/T6	Larva	Kilpisjärvi, Finland, 23.viii.1998	T. Nyman
	*gemmahelveticae*	*helvetica*	04363/T7	Larva	Vent, Austria, 4.viii.2004	T. Nyman
	*myrsinifoliae*	*myrsinifolia*	98371/T8	Larva	Kilpisjärvi, Finland, 23.viii.1998	T. Nyman

*Euura *(Shoot gall)	*atra*	*alba*, *fragilis*	97005/T9	♀, *ex pupa*	Joensuu, Finland, 30.v.1997	T. Nyman
	*eleagnos*	*eleagnos*	04357/TX	Larva	Pertisau, Austria, 13.viii.2004	T. Nyman
	*lapponica*	*lapponum*	98329/V1	Larva	Kilpisjärvi, Finland, 19.viii.1998	T. Nyman

*Euura *(Petiole gall)	*venusta*	*aurita*, *caprea*, *cinerea*	00038/V3	Larva	Joensuu, Finland, 20.viii.2000	T. Nyman

Outgroups						

*Phyllocolpa *(Leaf fold/roll)	*carinifrons*^1^	*pentandra*	98120/C2	Larva	Kesälahti, Finland, 16.vii.1998	H. Roininen
	*anomaloptera*^2^	*caprea*	97097/X9	Larva	Joensuu, Finland, 18.vii.1997	T. Nyman
	*alienata*^3^	*aurita*, *cinerea*	04055/X5	Larva	Nymindegab, Denmark, 17.vi.2004	T. Nyman
	sp. (near *tuberculata*)	*starkeana*	97063/E9	Larva	St. Petersburg, Russia, 15.vi.1997	A. Zinovjev

*Nematus *(External feeder)	*melanaspis*	*pentandra*	01092/D6	♀, *ex larva*	Parikkala, Finland, 24.vi.2001	T. Nyman

In the case of oligophagous species, the host of the sampled individual is underlined

^1^Species name according to recent revision [33], previously named *P. excavata*.

^2^Species name according to recent revision [34], previously named *P. leucapsis*.

^3^Species name according to recent revision [35], previously named *P. coriacea*.

### Phylogeny reconstruction

DNA sequence data was generated for 1528 base pairs of two mitochondrial genes (cytochrome oxidase I: 810 bp; cytochrome *b*: 718 bp) using protocols described previously [18,25]. All sequences have been deposited in GenBank under accession numbers DQ302205, DQ302212, and EU083911–EU084004. Modeltest 3.5 [27] was used in conjunction with PAUP* 4.0b10 [28] to select the GTR+I+Γ_4 _substitution model subsequently implemented in a Bayesian phylogenetic analysis in MrBayes 3.1.1 [29]. Two parallel runs employing default priors and consisting of four incrementally heated chains (t = 0.2) were run for six million generations while sampling trees from the current cold chain every 100 generations, and 110002 post-burn-in trees were used to calculate a Bayesian consensus tree. Because the tree is strongly supported and maximum-parsimony and maximum-likelihood analyses of the sequence data led to near-identical results, only the Bayesian tree was used in the permutation tests described below.

### Character evolution analyses and statistical tests

Ancestral gall types were inferred by accelerated transformations parsimony optimization [30], and ancestral gall locations (leaf, shoot, bud, or petiole) by maximum likelihood (ML) reconstruction in Mesquite 1.11 [31]. In the ML analysis, we employed an Mk1 model (= equal forward and reverse rates) after pruning the external-feeding outgroup from the galler tree. Because maximum rates of parasitism by different enemy species are not independent in galler population samples, we treated species-level enemy communities (= rows in the table in Additional file 1) as the observation for each galler species, and then devised three alternative permutations of enemy communities over galler species to test correlations between the parasitism matrix and the galler phylogeny. If parasite communities tend to be similar among closely related galler species, the length of the enemy data, as calculated on the basis of the galler topology, will be shorter than expected from random assignment. Data length [32] was calculated first from the observed data, and subsequently from 10000 random permutations of enemy communities over galler species. The probability that by chance data is as short as observed is calculated by comparing the observed data length with the distribution of permuted data lengths. Possible phylogenetic correlations within gall types were tested by restricting the permutations to occur only among sawfly species that induce similar galls.

To test whether an effect of the willow host species extends across gall-type boundaries, we used a paired replacement procedure: the enemy community of a randomly chosen galler species was replaced with the enemy communities of two randomly selected species. Those two species had the same gall type (different from that of the species being replaced) but only one of those two had a different host plant. The other species thus had the same host plant as the species being replaced. The absolute change in data length was calculated for each of the two replacements, and subsequently the difference in data-length change was calculated between the two replacements. If the replacement with a species that shares the host plant with the species being replaced results in a smaller absolute change in data length, the difference in absolute data length change becomes negative (and conversely, positive if the species not sharing the host plant yields a smaller data-length change). The distribution of differences in absolute tree-length change resulting from 10000 such paired replacements was evaluated against the null hypothesis (= mean change in absolute data length equal) using a paired samples *t *test. For the purposes of the species effect tests, willows belonging to two species complexes consisting of sister taxa with extensive hybridization (*S. alba *and *S. fragilis*; and *S. caprea*, *S. aurita*, and *S. cinerea*) were synonymized under two "superspecies". All permutation and replacement tests were performed in Matlab (The MathWorks, Inc., 3 Apple Hill Drive, Natick, MA 01760-2098, USA), see Additional file 2 for the scripts used for the tests. ANCOVA tests used for testing factors influencing numbers of associated parasitoid species and rates of parasite-inflicted mortality were performed using SPSS for Windows 14.0 (SPSS, Inc., 233 S. Wacker Drive, Chicago, IL 60606-6307, USA).

## Authors' contributions

TN was responsible for research planning, sequencing, phylogeny reconstruction, and writing. Statistical tests were devised by TN and FB and programmed by FB. Galler population sampling and enemy rearings were performed by JPK. All authors participated in the writing process. All the authors have read and approved the final manuscript.

## Supplementary Material

Additional file 1 Excel file showing maximum rates of mortality inflicted by each inquiline and parasitoid species on each galler species. Click here for file

Additional file 2 PDF file giving scripts used to perform permutation and replacement tests in Matlab Click here for file

## References

[B1] Singer MS, Stireman JO (2005). The tri-trophic niche concept and adaptive radiation of phytophagous insects. Ecol Lett.

[B2] Kitching RL (2006). Crafting the pieces of the diversity jigsaw puzzle. Science.

[B3] Ehrlich PR, Raven PH (1964). Butterflies and plants: a study in coevolution. Evolution.

[B4] Strong DR, Lawton JH, Southwood TRE (1984). Insects on Plants: Community Patterns and Mechanisms.

[B5] Novotny V, Drozd P, Miller SE, Kulfan M, Janda M, Basset Y, Weiblen GD (2006). Why are there so many species of herbivorous insects in tropical rainforests?. Science.

[B6] Farrell BD, Dussourd DE, Mitter C (1991). Escalation of plant defense: do latex and resin canals spur plant diversification?. Am Nat.

[B7] Farrell BD (1998). "Inordinate fondness" explained: why are there so many beetles?. Science.

[B8] Vamosi SM (2005). On the role of enemies in divergence and diversification of prey: a review and synthesis. Can J Zool.

[B9] Price PW, Bouton CE, Gross P, McPheron BA, Thompson JN, Weis AE (1980). Interactions among three trophic levels: influence of plants on interactions between insect herbivores and natural enemies. Annu Rev Ecol Syst.

[B10] Hawkins BA (1994). Pattern and Process in Host-Parasitoid Interactions.

[B11] Janz N, Nylin S, Wahlberg N (2006). Diversity begets diversity: host expansions and the diversification of plant-feeding insects. BMC Evol Biol.

[B12] Abrahamson WG, Blair CP, Eubanks MD, Morehead SA (2003). Sequential radiation of unrelated organisms: the gall fly *Eurosta solidaginis *and the tumbling flower beetle *Mordellistena convicta*. J Evol Biol.

[B13] Stireman JO, Nason JD, Heard SB, Seehawer JM (2006). Cascading host-associated genetic differentiation in parasitoids of phytophagous insects. Proc R Soc Lond B.

[B14] Lill JT, Marquis RJ, Ricklefs RE (2002). Host plants influence parasitism of forest caterpillars. Nature.

[B15] Murphy SM (2004). Enemy-free space maintains swallowtail butterfly host shift. Proc Natl Acad Sci USA.

[B16] Price PW, Fernandes GW, Waring GL (1987). Adaptive nature of insect galls. Environ Entomol.

[B17] Stone GN, Schönrogge K (2003). The adaptive significance of insect gall morphology. TREE.

[B18] Nyman T, Widmer A, Roininen H (2000). Evolution of gall morphology and host-plant relationships in willow-feeding sawflies (Hymenoptera: Tenthredinidae). Evolution.

[B19] Roininen H, Nyman T, Zinovjev AG, Raman A, Schaefer CW, Withers TM (2005). Biology, ecology, and evolution of gall-inducing sawflies (Hymenoptera: Tenthredinidae and Xyelidae). Biology, Ecology, and Evolution of Gall-inducing Arthropods.

[B20] Nyman T, Farrell BD, Zinovjev AG, Vikberg V (2006). Larval habits, host-plant associations, and speciation in nematine sawflies (Hymenoptera: Tenthredinidae). Evolution.

[B21] Kopelke J-P (1999). Gallenerzeugende Blattwespen Europas – Taxonomische Grundlagen, Biologie und Ökologie (Tenthredinidae: Nematinae: *Euura*, *Phyllocolpa, Pontania*). Cour Forsch-inst Senckenb.

[B22] Kopelke J-P (2003). Natural enemies of gall-forming sawflies on willows (*Salix *spp.). Entomol Gener.

[B23] Thompson JN (1999). Specific hypotheses on the geographic mosaic of coevolution. Am Nat.

[B24] Craig TP, Itami JK, Horner JD (2007). Geographic variation in the evolution and coevolution of a tritrophic interaction. Evolution.

[B25] Nyman T, Zinovjev AG, Vikberg V, Farrell BD (2006). Molecular phylogeny of the sawfly subfamily Nematinae (Hymenoptera: Tenthredinidae). Syst Entomol.

[B26] Pennacchio F, Strand MR (2006). Evolution of developmental strategies in parasitic Hymenoptera. Annu Rev Entomol.

[B27] Posada D, Crandall KA (1998). Modeltest: testing the model of DNA substitution. Bioinformatics.

[B28] Swofford DL (2002). PAUP* Phylogenetic Analysis Using Parsimony (*and Other Methods), version 40b10.

[B29] Ronquist F, Huelsenbeck JP (2003). MrBayes 3: Bayesian phylogenetic inference under mixed models. Bioinformatics.

[B30] Swofford DL, Maddison WP (1987). Reconstructing ancestral character states under Wagner parsimony. Math Biosci.

[B31] Maddison WP, Maddison DR (2006). Mesquite: a modular system for evolutionary analysis, version 1.11. http://mesquiteproject.org.

[B32] Pauplin Y (2000). Direct calculation of a tree length using a distance matrix. J Mol Evol.

[B33] Kopelke J-P The species of the *Phyllocolpa leucosticta*-group in Europe (Hymenoptera: Tenthredinidae: Nematinae). Senckenb Biol.

[B34] Kopelke J-P The species of the *Phyllocolpa crassispina*-, *scotaspis*-, and *piliserra*-group in Europe (Hymenoptera: Tenthredinidae: Nematinae). Senckenb Biol.

[B35] Kopelke J-P The species of the *Phyllocolpa leucapsis*-group in Europe (Hymenoptera: Tenthredinidae: Nematinae). Senckenb Biol.

