# Supplementary material for: Reciprocal diversification in a complex plant-herbivore-parasitoid food web
Source: BMC Biol. 2007 Nov 1;5:49. doi: 10.1186/1741-7007-5-49 (PMC2203972; doi:10.1186/1741-7007-5-49)
Supplement: Additional file 2 — PDF file giving scripts used to perform permutation and replacement tests in Matlab [file 1741-7007-5-49-S2.pdf]

Scripts 1. Matlab scripts used in the permutation and replacement tests.

```
function y = Tommi(datafiles, nsims,permtype,pa);
%returns #_simulations x 1 or #_simulations x 3 array of data lengths y,
%where the data has been randomized, randomized within host groups,
%randomized within gall groups, or randomized in a special fashion
%to test for an effect of host plant.
% y(1,1) is always the tree length of the original data!
%INPUT:
%nsims: number of permutations. permtype: type of permutation
%permtype == empty : full randomizations
%other options; permtype == hostspecific / gallspecific / hosteffect.
%pa: use presence-absence (pa==1) or quantitative(pa==0) data.
%NOTE: one has to change the filenames below for the code to work.

data = load('tommidata.txt'); %parasite data
data = data; %ensure matrix dimensions
if pa
    data(find(data))=1; %converts matrix to presence-absence
end
ordata = data; %copy of original configuration
galls = load('tommigalls.txt'); %gall type data
Tree = phytreeread('tommitree.txt'); %phylogenetic tree
ns = size(data,1); %number of galler species
np = size(data,2); %number of parasites
fid = fopen('tommihosts.txt', 'r'); %open host file
hosts = textscan(fid, '%s '); %read data
fclose(fid); %close file
hosts = cellstr(hosts{:}); %convert to cell array
[uh mh nh] = unique(hosts); %find unique hosts
[ug mg ng] = unique(galls); %find unique galls

%Create matrix B of connecting branch numbers
[pd,C] = pdist(Tree,'squareform',true); %returns in C the index of the closest common parent
nodes for every possible pair of query nodes.
P = get(Tree,'Pointers'); %parent-child pointers
for j=2:ns
    for k=1:j-1
        B(j,k) = traceAB(j,C(j,k)) + traceAB(k,C(j,k));
        B(k,j) = B(j,k);
    end
end

for mc=1:nsims
    y(1,mc) = GetTreeLength; %store for output

    if strcmp(permtype,'gallspecific') %gall-specific permutation
        for g=1:length(ug) %for all unique hosts
            gg = find(ng==g); %indices of species with this host
            rp = randperm(length(gg)); %permutation vector for focal species
            data(gg,:) = ordata(gg(rp),:); %shuffle species with this host
        end
    elseif strcmp(permtype,'hostspecific') %host-specific permutation
        for h=1:length(uh) %for all unique hosts
            hh = find(nh==h); %indices of species with this host
            rp = randperm(length(hh)); %permutation vector for focal species
            data(hh,:) = ordata(hh(rp),:); %shuffle species with this host
        end
    elseif strcmp(permtype,'hosteffect') %test of host effect
        sgdhR2 = [];
        while isempty(sgdhR2)
            shdgR1 = [];
```

```
            while isempty(shdgR1)
                R1 = round(1+rand*(length(galls)-1)); %randomly select individual
                shR1 = find(nh==nh(R1)); %(indices of) species with same host as R1
                dgR1 = find(ng~=ng(R1)); %species with different gall than R1
                shdgR1 = intersect(shR1,dgR1); %species with same host but different gall
            end
            R2 = shdgR1(round(1+rand*(length(shdgR1)-1))); %R2: select species with same host
but different gall
            sgR2 = find(ng==ng(R2)); %species with same gall as R2
            dhR2 = find(nh~=nh(R2)); %species with different host than R2
            sgdhR2 = intersect(sgR2,dhR2); %species with same gall but different host
        end
        R3 = sgdhR2(round(1+rand*(length(sgdhR2)-1))); %R3: select species with same gall type
as R2, but different host
        data = ordata;
        y(1,mc) = y(1,1); %length using original data
        data(R1,:) = ordata(R2,:);
        y(2,mc) = GetTreeLength; %store for output
        data(R1,:) = ordata(R3,:);
        y(3,mc) = GetTreeLength; %store for output
        R3 = R3;
    else % normal permutation
        rp = randperm(43);
        data = ordata(rp,:);
    end

end
y=y'; %transpose results for easy viewing

    function nb = traceAB(a,b)
        %traces from child a to parent b and returns number of branches
        nb = 0; %number of branches
        q=a;
        while a~=b
            ind = find(P==q); %index of q as child
            [row col] = ind2sub(size(P), ind); %retrieve row, i.e. internal node
            nb = nb+1;
            if row==b
                break%parent encountered
            else
                q = ns+row; %continue search
            end
        end
    end %traceAB

    function Sdc = GetTreeLength(); %returns length of the current tree
        %calculate distances Dij
        for j=2:ns
            for k=1:j-1
                D(j,k) = sum(abs(data(j,:)-data(k,:))); %distance between species j and k
                D(k,j)=D(j,k);
            end
        end
        %calculate tree length Sdc
        Sdc=0;
        for j=1:ns
            for k=1:ns
                if k~=j
                    Sdc = Sdc + D(j,k)/2^B(j,k);
                end
            end
        end
    end%GetTreeLength
end
```
